# Association of arterial stiffness and central hemodynamics with moderately reduced glomerular filtration rate in Chinese middle-aged and elderly community residents: a cross-sectional analysis

**DOI:** 10.1186/s12882-018-0897-2

**Published:** 2018-05-02

**Authors:** Shihui Fu, Yi Guo, Leiming Luo, Ping Ye

**Affiliations:** 1 Department of Geriatric Cardiology, Chinese People’s Liberation Army General Hospital, Beijing, 100853 China; 2 Department of Cardiology and Hainan Branch, Chinese People’s Liberation Army General Hospital, Beijing, 100853 China

**Keywords:** Carotid-femoral pulse wave velocity, Central pulse pressure, Moderately reduced glomerular filtration rate

## Abstract

**Background:**

Kidney impairment constitutes severe risk for cardiovascular disease, stroke and all-cause mortality, and early identification and prevention of kidney impairment is critical to effective management of prognostic risk in community residents. Previous studies have validated that carotid-femoral pulse wave velocity (cfPWV) is a significant factor associated with chronic kidney disease. However, whether cfPWV is associated with moderately reduced glomerular filtration rate (GFR) remains unclear. This analysis was designed to examine the association of moderately reduced GFR with cfPWV and central pulse pressure (cPP) in Chinese middle-aged and elderly community residents.

**Methods:**

There were 875 community residents enrolled in this analysis, and then cfPWV and cPP were assessed in all participants following the standard procedure.

**Results:**

Entire cohort had a median (range) age of 66 (45–88) years, and 65.4% were women. Both cfPWV and cPP differed significantly between participants with and without moderately reduced GFR (*P* < 0.05 for all). Logistic regression analyses indicated that cfPWV and cPP had the significant association with moderately reduced GFR (*P* < 0.05 for all).

**Conclusion:**

This analysis demonstrated the significant association of cfPWV and cPP with moderately reduced GFR in Chinese middle-aged and elderly community residents.

## Background

Kidney impairment constitutes severe risk for cardiovascular disease, stroke and all-cause mortality, and early identification and prevention of kidney impairment are critical to effective management of prognostic risk in community residents. Recently, more and more attention has been given to the association of arterial stiffness and central hemodynamics with kidney impairment, especially early kidney impairment [1]. Previous studies have validated that carotid-femoral pulse wave velocity (cfPWV) can serve as not only a noninvasive measure of central artery stiffness, but also a significant factor associated with chronic kidney disease (CKD) [2–4]. However, whether cfPWV is associated with moderately reduced glomerular filtration rate (GFR) remains unclear, especially in Chinese middle-aged and elderly community residents. Further studies are needed to assess the association between cfPWV and moderately reduced GFR.

Several reports have indicated the evidence of CKD present in patients with abnormal central hemodynamics indicated by elevated central pulse pressure (cPP) [5–7]. Moreover, recent study has suggested that cPP rather than peripheral pulse pressure (pPP) is reflective of CKD [8]. However, whether both cPP and pPP are closely associated with moderately reduced GFR is unclear, especially in Chinese middle-aged and elderly community residents. Thus, this analysis was designed to: 1) assess the association between cfPWV and moderately reduced GFR; and 2) observe the cPP and pPP in their association with moderately reduced GFR in Chinese middle-aged and elderly community residents.

## Methods

### Study population

There were 937 participants aged 45 years and older in this analysis. According to the stratified cluster sampling design of health check-up program from May 2007 to July 2009, all participants were permanent residents of four communities in three districts (Fengtai, Shijingshan and Daxing) of Beijing, China. Of those participants, 62 participants had GFR below 60 ml/min/1.73 m^2^. Therefore, this analysis had 875 participants. This analysis was approved by Ethics Committee of Chinese People’s Liberation Army General Hospital (Beijing, China). Written consent was obtained from each participant.

### Arterial stiffness and hemodynamics

Following the standard procedures, peripheral blood pressure was measured on brachial artery with mercury sphygmomanometer (Yuwell medical equipment & supply Co., Ltd., Jiangsu, China). Blood pressure was measured in duplicate and averaged. Pulse pressure (PP) was calculated as systolic blood pressure minus diastolic blood pressure. Participants with systolic blood pressure ≥ 140 mmHg, diastolic blood pressure ≥ 90 mmHg or hypotensive drugs were regarded to have hypertension. Arterial stiffness was evaluated by a device designed for automatic cfPWV analysis (Createch Industrie, Garges les Gonesse, France). Radial artery pressure waveform was evaluated by a device designed for automatic pulse wave analysis (SphygmoCor, Sydney, Australia), and the corresponding cPP was calculated with its transfer function. Their technical characteristics have been described previously [9].

### Laboratory examination

Blood sample was drawn in the morning after the overnight fasting and examined by central laboratory on the same date. Concentrations of fasting plasma glucose (FPG), triglyceride, high-density lipoprotein-cholesterol (HDL-c), low-density lipoprotein-cholesterol (LDL-c) and serum creatinine were examined with enzymatic assays (Roche Products Ltd., Basel, Switzerland). Diabetes mellitus referred to those with FBG ≥ 7.0 mmol/L or hypoglycemic treatment. Chinese modified Modification of Diet in Renal Disease equation was used as an evaluation of GFR: 175 × serum creatinine (mg/dL)^-1.234^ × age (year)^-0.179^ × 0.79 (if female) [10]. Range of moderately reduced GFR was from 60 mL/min/1.73m^2^ to 89 mL/min/1.73m^2^. Laboratory examination was completed by qualified technicians without knowledge of clinical information.

### Statistical analysis

Statistical analysis was implemented with Statistical Package for the Social Science version 17 (SPSS, Inc., Chicago, IL, USA). Categorical data were reported as number and percentage. Continuous data were reported as mean and standard deviation for variables with a normal distribution, and median and interquartile range for non-normally distributed variables. Difference between groups was assessed by Student’s t-test for continuous data with a normal distribution, Mann–Whitney U test for non-normally distributed data and x^2^ test for categorical data. Pearson’s correlation for continuous variables with normal distribution and Spearman’s correlation for categorical variables were used to evaluate their simple correlation of GFR with cfPWV and PP. Logistic regression analysis (Enter) was used to evaluate multivariate association of cfPWV and PP with moderately reduced GFR after adjustment of age, HDL-c and LDL-c. Age, HDL-c and LDL-c, rather than gender, hypertension, diabetes mellitus, FPG and triglyceride, were significant in univariate analysis with moderately reduced GFR, and included as the adjustment of multivariate analysis with moderately reduced GFR. Two-sided *P* value < 0.05 was accepted as significant.

## Results

Entire cohort had a median (range) age of 66 (45–88) years, and 65.4% (572 participants) were women. Prevalence of hypertension and diabetes mellitus were 52.7% (461 participants) and 24.3% (213 participants), respectively. Characteristics of the study cohort with and without moderately reduced GFR were depicted in Table 1. Age, HDL-c and LDL-c were significantly different between participants with and without moderately reduced GFR (*P* < 0.05 for all). cfPWV differed significantly between participants with and without moderately reduced GFR (P < 0.05). Both cPP and pPP had the significant difference between participants with and without moderately reduced GFR (*P* < 0.05 for all).

**Table 1:** Characteristics of the study cohort with and without moderately reduced GFR

Characteristics	Normal GFR (*n* = 370)	moderately reduced GFR (*n* = 505)	*P* value
Age (year)^a^	65(60–70)	67(62-72)	< 0.001
Males (%)	121(32.7)	182(36.0)	0.305
Hypertension (%)	184(49.7)	277(54.9)	0.134
Diabetes mellitus (%)	102(27.6)	111(22.0)	0.057
pPP (mmHg)^a^	50(40–60)	55(46-65)	< 0.001
FPG (mmol/L)^a^	5.1(4.4-5.8)	5.0(4.4–5.8)	0.969
Triglyceride (mmol/L)^a^	1.5(1.2-2.2)	1.5(1.2–2.1)	0.616
HDL-c (mmol/L)^a^	1.4(1.1-1.6)	1.3(1.1–1.6)	0.030
LDL-c (mmol/L)^a^	3.0(2.5-3.4)	3.1(2.6–3.5)	0.014
GFR (ml/min/1.73 m^2^)^a^	101.6(94.5–113.9)	80.1(74.2–85.2)	< 0.001
cfPWV (m/s)^a^	11(10–13)	12(10-15)	< 0.001
cPP (mmHg)^a^	42(35–51)	46(38-56)	< 0.001

^a^median (interquartile range)

*GFR* glomerular filtration rate, *pPP* peripheral pulse pressure, *FPG* fasting plasma glucose, *HDL-c* high-density lipoprotein-cholesterol, *LDL-c* low-density lipoprotein-cholesterol, *cfPWV* carotid-femoral pulse wave velocity, *cPP* central pulse pressure

**Fig. 1:**
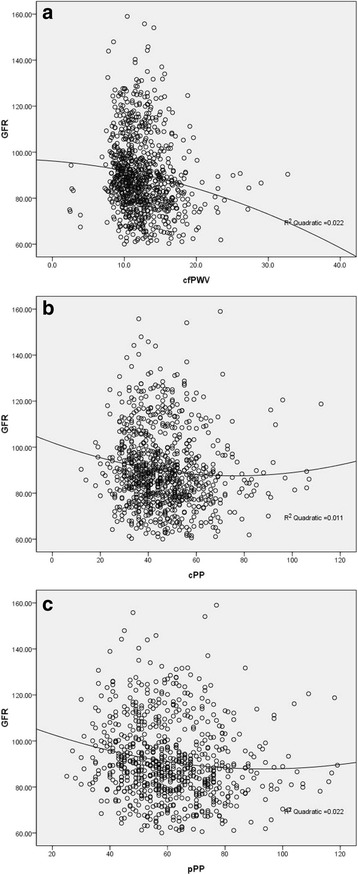
**a** scatter plot between GFR and cfPWV; **b** scatter plot between GFR and cPP; **c** scatter plot between GFR and pPP. GFR: glomerular filtration rate; cfPWV: carotid-femoral pulse wave velocity; cPP: central pulse pressure; pPP: peripheral pulse pressure

Either two of cfPWV, cPP and pPP were significantly correlated with each other (*P* < 0.001 for all; cfPWV and cPP: *r* = 0.368; cfPWV and pPP: *r* = 0.374; cPP and pPP: *r* = 0.797) (See Fig. 1). GFR was significantly correlated with age, HDL-c, LDL-c, cfPWV, cPP and pPP (*P* < 0.05 for all). Simple correlation and multivariate association of cfPWV and PP with moderately reduced GFR were shown in Table 2. In Logistic regression analyses, age, HDL-c and LDL-c were significantly associated with moderately reduced GFR (*P* < 0.05 for all). cfPWV had the significant association with moderately reduced GFR (P < 0.05). There was the significant association of cPP and pPP with moderately reduced GFR (*P* < 0.05 for all).

**Table 2:** Association of cfPWV and PP with moderately reduced GFR in Logistic regression analyses

Variables		r value	*P* value	HR value (95%CI)	*P* value
cfPWV model	Age (year)	−0.195	< 0.001	1.026(1.004–1.048)	0.018
	HDL-c (mmol/L)	0.101	0.003	0.682(0.479–0.970)	0.033
	LDL-c (mmol/L)	−0.080	0.017	1.237(1.023–1.494)	0.028
	cfPWV (m/s)	−0.179	< 0.001	1.066(1.013–1.121)	0.014
cPP model	Age (year)	−0.195	< 0.001	1.027(1.006–1.049)	0.011
	HDL-c (mmol/L)	0.101	0.003	0.685(0.482–0.974)	0.035
	LDL-c (mmol/L)	−0.080	0.017	1.238(1.024–1.495)	0.027
	cPP (mmHg)	−0.132	< 0.001	1.015(1.004–1.026)	0.009
pPP model	Age (year)	−0.195	< 0.001	1.030(1.009–1.051)	0.004
	HDL-c (mmol/L)	0.101	0.003	0.687(0.483–0.977)	0.037
	LDL-c (mmol/L)	−0.080	0.017	1.242(1.027–1.501)	0.025
	pPP (mmHg)	−0.172	< 0.001	1.012(1.003–1.022)	0.012

*cfPWV* carotid-femoral pulse wave velocity, *PP* pulse pressure, *GFR* glomerular filtration rate, *HR*hazard ratio, *CI* confidence interval, *cPP* central pulse pressure, *pPP* peripheral pulse pressure, *HDL-c* high-density lipoprotein-cholesterol, *LDL-c* low-density lipoprotein-cholesterol

## Discussion

This analysis had two main findings in Chinese middle-aged and elderly community residents: firstly, cfPWV was significantly associated with moderately reduced GFR; secondly, both cPP and pPP had the significant association with moderately reduced GFR.

cfPWV is a noninvasive representative of central arterial stiffness [2, 4]. In previous studies, cfPWV has been shown to be closely linked to CKD [1, 3]. There were the scarce and controversial studies appraising the relationship of cfPWV with moderately reduced GFR, and it is necessary to determine the association of cfPWV with moderately reduced GFR, especially in Chinese middle-aged and elderly community residents [11]. This analysis confirmed that the significant association existed between cfPWV and moderately reduced GFR.

Central and peripheral hemodynamics are commonly assessed by cPP and pPP, respectively. Previous researches have realized that increased cPP rather than pPP correlated with reduced kidney blood flow and GFR in patients with CKD, and even in those after kidney transplantation [5–8]. However, it is uncertain for the association of cPP and pPP with moderately reduced GFR, especially in Chinese middle-aged and elderly community residents. This analysis found that both cPP and pPP were significantly associated with moderately reduced GFR in Chinese middle-aged and elderly community residents. Association of moderately reduced GFR with cfPWV, cPP and pPP was all significant in this analysis, and future studies are needed to evaluate the relative value of theses methods in evaluating arterial function.

## Conclusion

This analysis demonstrated the significant association between cfPWV and moderately reduced GFR. Meanwhile, both cPP and pPP were significantly associated with moderately reduced GFR in Chinese middle-aged and elderly community residents.

## Abbreviations

cfPWV: carotid-femoral pulse wave velocity
CKD: chronic kidney disease
cPP: central pulse pressure
FPG: fasting plasma glucose
GFR: glomerular filtration rate
HDL-c: high-density lipoprotein-cholesterol
LDL-c: low-density lipoprotein-cholesterol
pPP: peripheral pulse pressure
SPSS: Statistical Package for the Social Science
